# Regulation and targeting of enzymes mediating Parkinson's disease pathogenesis: focus on Parkinson's disease kinases, GTPases, and ATPases

**DOI:** 10.3389/fnmol.2014.00071

**Published:** 2014-07-29

**Authors:** Jean-Marc Taymans, Veerle Baekelandt, Kirsten Harvey

**Affiliations:** ^1^Laboratory for Neurobiology and Gene Therapy, Department of NeurosciencesKU Leuven, Leuven, Belgium; ^2^School of Pharmacy, University College LondonLondon, UK

**Keywords:** LRRK2, ATP13A2, PINK1, tau proteins, alpha-Synuclein, ROCO proteins, phosphorylation

Understanding the molecular pathogenesis of Parkinson's disease (PD) is a priority area in biomedical research. It is a pre-requisite to developing disease-modifying strategies and to improve early diagnosis of disease. Over the last two decades, geneticists have identified several genes underlying PD. Some of these gene changes segregate with Mendelian forms of the disease. Others were identified as genetic risk factors of sporadic PD via genome-wide association studies (GWAS).

Interestingly, a number of PD proteins have enzymatic functions, including kinase, GTPase or ATPase activity. As enzymes are often key elements in the regulation of cellular signaling networks, they themselves or additional pathway components might provide new therapeutic targets for disease modifying therapies. For instance, kinases mediate phosphorylation events, which activate or inactivate their substrates, while GTPases modulate activity of their effector proteins via direct interaction in a GDP/GTP dependent manner. ATPases also control cellular processes through their involvement in cellular energy production and/or in transmembrane transport. Finally, as is typical in cellular signaling networks, enzymes are themselves not generally constitutively active, but rather they are subject to regulation. Knowledge of how PD kinases, GTPases, and ATPases are activated or inactivated can aid in understanding how signaling networks are deregulated in PD and can also point to new therapeutic targets. This special Research Topic of *Frontiers in Molecular Neuroscience* discusses new key knowledge on the regulation and targeting of kinases, GTPases and ATPases in PD and presents new original research in this field.

The PD enzymes discussed in this Research Topic were discovered either in genetic linkage or genetic association studies (www.genenames.org/genefamilies/PARK, www.pdgene.org, Lill et al., 2012) or in biological studies revealing a strong functional link with one of the PD proteins. In this context, PD enzymes are the ATPase ATP13A2 encoded by a gene at the PARK9 locus, several GTPases, including the small GTPase RAB7L1, and leucine-rich repeat kinase 2 (LRRK2) which functions as a kinase and GTPase (Greggio, 2012; Taymans, 2012). Several PD proteins are kinases including Pten-induced kinase 1 (PINK1) and LRRK2, discovered in genetic linkage studies in familial PD patients. In addition, LRRK2 and cyclin G associated kinase (GAK) were identified in genetic association studies in sporadic PD sufferers. Importantly, the pathogenicity of some gene products of PD genes is also regulated by phosphorylation by upstream kinases, such as polo-like kinase 2 (PLK2), which phosphorylates the PD protein α-synuclein. There are also multiple kinases phosphorylating the microtubule associated protein tau (MAPT), a protein known to be involved in Alzheimer's disease and associated to PD in GWAS (Lill et al., 2012).

Van Veen et al. review the cellular function and pathological role of ATP13A2 and related P-type transport ATPases in PD and other neurological disorders (Van Veen et al., 2014). P-type ATPases are transmembrane transporters, which use energy from ATP hydrolysis to transport substrates across membranes and against their concentration gradients. The authors provide an overview of this family of proteins and their molecular and biochemical features. An overview is also given of the involvement of P-type ATPases in multiple neurological disorders. They specifically elaborate on ATP13A2, which causes a severe form of early onset Parkinsonism known as Kufor-Rakeb syndrome.

Rivero-Ríos et al. review the evidence pointing to the upstream deregulation of calcium signaling in Parkinson's disease (Rivero-Ríos et al., 2014). Ca^2+^ is not only an important secondary messenger in cellular signaling, but is also essential for the proper functioning of multiple cellular organelles. The authors discuss the role of PD genes in the regulation of Ca^2+^ homeostasis as well as the potential therapeutic strategies to slow PD progression based on counteracting abnormal Ca^2+^ handling.

When studying kinases involved in disease processes, a common pathomechanism is hyperphosphorylation of substrates. In their review, Tenreiro et al. explore the phosphorylation of two important proteins in PD, namely α-synuclein and tau (Tenreiro et al., 2014). A-synuclein is a key protein in PD, as it is a main component of the defining pathological feature of PD, Lewy bodies, and genetic variants of α-synuclein are linked with familial PD and associated with sporadic PD. Interestingly, phosphorylation of one key residue (serine 129) is associated with PD pathology. By contrast, tau contains multiple phosphorylation sites associated with disease, and is known to be involved in AD, although it also contributes to some forms of Parkinsonism. Another common feature of cellular functioning of kinases is that often multiple kinases functionally interact with each other in signaling networks. Related to this, Matenia and Mandelkow discuss recent work revealing an association of PINK1 with the microtubule associated kinase MARK2 which is involved in axonal transport (Matenia and Mandelkow, 2014).

Arguably, LRRK2 is one of the most important proteins involved in PD, and this is reflected by the number of papers on LRRK2 in this Research Topic. Gilsbach and Kortholt review the available structural biology data on homologs of LRRK2 (Gilsbach and Kortholt, 2014). The structure of other members of this Ras of complex proteins (ROCO) family has potential implications for LRRK2 functioning. Indeed, ROCO proteins from lower organisms such as bacteria and slime molds have yielded atomic structure data, which has begun to reveal mechanistic insight in the functioning of this poorly known family of proteins. The authors focus on structures of the ROCO GTPase and kinase domains. Although there is a good degree of structural and functional heterogeneity between different ROCO proteins, the recently elucidated ROCO structures are suggestive of common mechanisms for ROCO proteins, which can be tested for LRRK2 in the future. In the absence of structures for human LRRK2, the combination of these data with molecular models of LRRK2 (such as the optimized human LRRK2 kinase domain model presented in another paper of this issue, Vancraenenbroeck et al., 2014) can provide insight into the structural underpinnings of LRRK2 functions.

Boon et al. (2014) discuss aspects of LRRK2 as a regulator of cellular signaling, with a focus on reported interactions of LRRK2 with MAPK signaling cascades, GTPases and GTPase regulating proteins. This paper also discusses the key issues in LRRK2 biology to link biochemical activity of LRRK2 to cell biological effects (Boon et al., 2014). Related to this, Cirnaru et al. report original research showing that LRRK2 kinase activity regulates synaptic vesicle trafficking and neurotransmitter release through modulation of LRRK2 macro-molecular complexes (Cirnaru et al., 2014). The LRRK2 kinase activity is currently regarded as a potential therapeutic target, however several issues remain, most specifically in how to monitor this activity in cell culture. Related to this is the fact that LRRK2 is itself a phosphorylated protein (Gloeckner et al., 2010; Lobbestael et al., 2012). The LRRK2 phosphorylation sites are potential correlates for LRRK2 activity. Reynolds et al. (2014) provide an extensive study characterizing in parallel multiple autophosphorylation and cellular phosphorylation sites and explore how the regulation of these sites correlates to LRRK2 kinase activity and biology. Vancraenenbroeck et al. studied another aspect of LRRK2 cellular phosphorylation by using a kinome-wide panel of kinase inhibitors to identify compounds that regulate LRRK2 phosphorylation levels. This type of approach is designed to inhibit upstream kinases phosphorylating LRRK2 and compounds that lead to dephosphorylation are expected to point to these upstream kinases. The correlation of cellular activity with the *in silico* and *in vitro* analysis of the kinase inhibitors shows that the strongest dephosphorylation of LRRK2 is induced by compounds which act on LRRK2 itself, without excluding a role for upstream kinases (Vancraenenbroeck et al., 2014).

Kinases are a very attractive class of drug targets in general and much expertise has been built up from the development of kinase inhibitors for the treatment of cancers. Kinase inhibitors have a relatively low attrition rate in development compared to compounds directed toward other classes of targets and several kinase inhibitors are now in routine clinical practice, mostly in the field of oncology. Developing small compounds to modulate GTPase activities on the other hand is a path much less traveled. Given the number of potential GTPase targets for the treatment of PD, including LRRK2 (Taymans, 2012) or RAB7L1 (Beilina et al., 2014), approaches to discover and develop drugs modulating GTPases merit further investigation. In their review, Hong and Sklar (2014) discuss several GTPases which may be targeted for PD therapy and suggest approaches for GTPase drug discovery. Finally, to have a full overview of kinases involved in PD, it is important to have an understanding of their brain expression patterns. This subject is extensively reviewed by Dzamko et al. (2014) for a number of PD kinases, including LRRK2, GAK, and PLK2.

## Conflict of interest statement

The Associate Reviewer Patric Lewis declares that, despite being affiliated to the same institution as author Kirsten Harvey, the review process was handled objectively and no conflict of interest exists. The authors declare that the research was conducted in the absence of any commercial or financial relationships that could be construed as a potential conflict of interest.
